# Change in Allosteric Network Affects Binding Affinities of PDZ Domains: Analysis through Perturbation Response Scanning

**DOI:** 10.1371/journal.pcbi.1002154

**Published:** 2011-10-06

**Authors:** Z. Nevin Gerek, S. Banu Ozkan

**Affiliations:** 1 Center for Biological Physics, Arizona State University, Tempe, Arizona, United States of America; 2 Department of Physics, Arizona State University, Tempe, Arizona, United States of America; National Cancer Institute, United States of America and Tel Aviv University, Israel

## Abstract

The allosteric mechanism plays a key role in cellular functions of several PDZ domain proteins (PDZs) and is directly linked to pharmaceutical applications; however, it is a challenge to elaborate the nature and extent of these allosteric interactions. One solution to this problem is to explore the dynamics of PDZs, which may provide insights about how intramolecular communication occurs within a single domain. Here, we develop an advancement of perturbation response scanning (PRS) that couples elastic network models with linear response theory (LRT) to predict key residues in allosteric transitions of the two most studied PDZs (PSD-95 PDZ3 domain and hPTP1E PDZ2 domain). With PRS, we first identify the residues that give the highest mean square fluctuation response upon perturbing the binding sites. Strikingly, we observe that the residues with the highest mean square fluctuation response agree with experimentally determined residues involved in allosteric transitions. Second, we construct the allosteric pathways by linking the residues giving the same directional response upon perturbation of the binding sites. The predicted intramolecular communication pathways reveal that PSD-95 and hPTP1E have different pathways through the dynamic coupling of different residue pairs. Moreover, our analysis provides a molecular understanding of experimentally observed hidden allostery of PSD-95. We show that removing the distal third alpha helix from the binding site alters the allosteric pathway and decreases the binding affinity. Overall, these results indicate that (i) dynamics plays a key role in allosteric regulations of PDZs, (ii) the local changes in the residue interactions can lead to significant changes in the dynamics of allosteric regulations, and (iii) this might be the mechanism that each PDZ uses to tailor their binding specificities regulation.

## Author Summary

PDZ domain proteins (PDZs) act as adapters in organizing functional protein complexes. Through dynamic interactions, PDZs play a key role in mediating key cellular functions in the cell, and they are linked to currently challenging diseases including Alzheimer's, Parkinson's and cancer. Moreover, they are associated with allosteric regulations in mediating signaling. Therefore, it is critical to have knowledge of how the allosteric transition occurs in PDZs. We investigate the allosteric response of the two most studied PDZs, PSD-95 and hPTP1E, using the perturbation response scanning (PRS) approach. The method treats the protein as an elastic network and uses linear response theory (LRT) to obtain residue fluctuations upon exerting directed random forces on selected residues. With this efficient and fast approach, we identify the key residues that mediate long-range communication and find the allosteric pathways. Although the structures of PSD-95 and hPTP1E are very similar, our analysis predicts that their allosteric pathways are different. We also observe a significant change in allosteric pathways and a decrease in binding affinity upon removal of the distal α3 helix of PSD-95. This approach enables us to understand how dynamic interactions play an important role in allosteric regulations.

## Introduction

Allosteric regulation orchestrates functional behaviors in biological networks through appropriate switches. From a biochemical perspective, allostery can be described as a perturbation at one place in a protein structure, such as the binding of a ligand that alters the binding affinity of a distant site or enzymatic activity [1]. Several models have been suggested for explaining the ‘allosteric mechanism’. Models of conformational transition between co-existing states such as the MWC model of Monod [2], and the ‘induced fit’ KNF model of Koshland [3] were the first views among them. They described allostery as a binding event that causes conformational change via a single propagation pathway [4]. A new view of allosteric transitions supported from NMR studies, referred to as the ‘population shift’ model, has replaced the MWC and KNF models [5]–[8]. The population shift models claim that a protein in the unliganded form exhibits an ensemble of conformational states and ligand binding leads to a redistribution of the population of these states. In this view, it is important to explore how protein dynamics might contribute to allostery and make communication possible within a protein. Unlike the classical allostery models, the population shift-models also suggest that allostery can be mediated without any significant conformational change [9]–[15] but rather from changes in dynamics.

Moreover, recent experimental and theoretical evidences indicate that allostery is not limited to multi-domain proteins or complexes [5] and it may even be a fundamental property of all proteins, even single domain proteins. In single domain proteins, it is evident that residues that are energetically connected through structural rearrangements and dynamics lead to allosteric regulation [6], [11], [15]–[17]. More importantly, studies on single domain protein PDZ (post-synaptic density-95/discs large/zonula occludens-1) have indicated that allostery can arise not only from large conformational changes, but also from changes in dynamics [12], [14].

Indeed, PDZ domain proteins (PDZs) are the most studied system for understanding *single domain allostery* [11], [16], [18]–[25]. PDZs are small protein-protein interaction modules and typically recognize specific amino acids in the C-terminal end of peptide motifs or proteins [26]–[28]. Various studies on several PDZs, including statistical coupling analysis (i.e. sites that have correlated mutation based on evolutionary information) [16], [29], [30], molecular dynamics [11], [22], [31]–[33], normal mode analysis [34], [35], NMR relaxation methods and site directed mutational analysis [12], [18]–[20], [25], [36] have shown that several PDZs exhibit allosteric behavior that appears to connect incoming signals, notably binding to recognition motifs present on an upstream partner, to downstream partners [11], [16], [18]–[25]. In many different cellular contexts, PDZs function to transduce these binding events into favorable domain-domain assembly of complexes [14]. Thus, it is critical to understand the residues involved in these allosteric pathways in order to modulate the PDZ mediated interaction in cell regulation especially those in disease pathways. Moreover, a recent experimental study by Petit et al. [12], has confirmed yet another strong allosteric power of one of the PDZs: the hidden dynamic allostery. The removal of the non-canonical third helix (α3) in PSD-95 (PDZ3), which lies outside of the binding pocket, reduces the binding affinity drastically due to a change in side chain dynamics upon truncation, indicating the role of entropy and dynamics in allosteric regulation. More interestingly, further investigation has shown that the removal of this distal α3 disrupts the communication between PDZ3 and SH3-GK, which modulates the binding of Disc large protein (Dlg) to the localization protein GukHolder [37]. Therefore, the hidden dynamic allostery related with α3 is indeed a regulatory module within the context of larger interdomain interactions.

In summary, PDZs do not solely act as simple scaffold proteins. On the contrary through dynamics, they propagate signals to functionally important distant sites for intramolecular and intermolecular interactions [16]. They all have the same conserved structure and similar sequences [16], yet different PDZs have evolved different dynamics properties tailored to mediate different functions in the cell [14]. Thus, it would be very important to understand how signals are passed from one residue to another within the network of PDZs and how the sequential and structural variations alter the allosteric pathways for those allosteric PDZs [11], [18], [20]–[24]. Here we would like to tackle this problem with our new method called perturbation response scanning (PRS) [38], [39]. PRS treats the protein as an elastic network and uses linear response theory (LRT) to obtain residue fluctuations upon external perturbation. By sequentially exerting directed random forces on single residues along the chain of the unbound form and recording the resulting relative changes in the residue coordinates using LRT, we can successfully reproduce the residue displacements from the experimental structures of bound and unbound forms. The method is well established and tested for 25 proteins that display a variety of conformational motions upon ligand binding, including shear, hinge, allosteric, and partial refolding as well as more complex protein motions [39].

In the present study, we investigate the allosteric transitions by analyzing response fluctuation profiles upon perturbation on binding site residues by PRS. We focus on two widely studied PDZs: the third PDZ from the post-synaptic-density-95 (PSD-95 PDZ3) and the second PDZ from the human tyrosine phosphates 1E (hPTP1E PDZ2). The results from our computationally inexpensive and effective approach successfully identify the dynamically linked allosteric residues obtained from experiments (NMR or mutagenesis techniques) [12], [18]–[20], [25], [36] as well as evolutionarily coupled residues from sequence-based statistical approaches [16],[29],[30] and key residues predicted from molecular dynamics, normal mode analysis and protein energy-based networks [11], [22], [31]–[35], [40]. As a further test, we construct the communication pathway between these residues that might be responsible in transmitting allosteric signals. We achieve this through linking residues that show similar directionality of motion upon perturbation of binding sites. Interestingly, the constructed allosteric pathway indicates a strong structural residue coupling network. Moreover, we observe that the two PDZs, PSD-95 and hPTP1E, have distinct allosteric pathways despite their structural similarity, indicating the role of dynamic coupling in these domains [14], [35], [41]. The residues in the allosteric pathway of PSD-95 are homogenously distributed along the secondary structural motifs while the allosteric pathway of hPTP1E shows more localization around in regions of β1–β2 loop, β2 and β3 strands and the region of β5 strand and the α2 helix, missing the region of the α1 helix. The differences in the allosteric pathways of these two PDZs indicate the critical of role of dynamic coupling in PDZ domains and that differences in residue sequences within the same fold can lead to different dynamic coupling. Indeed, PDZs master this to mediate different cellular functions in different parts of the cell [14]. In addition to that, our PRS analysis indicates that the allosteric pathway of PSD-95 significantly alters upon removal of the distal third helix (α3 helix). This indicates that local changes in the network alter the directionalities of correlated motion, which may lead to a change in binding affinity [35], [42]. Strikingly, when we incorporate the change in backbone dynamics into the docking computation through generating multiple conformations by PRS, we also observe an increase in binding energies upon removal of the third helix.

## Results/Discussion

Our objective is to apply a computational approach, perturbation response scanning (PRS), to identify the network of dynamically important residues and propose a possible pathway responsible for intramolecular signaling. As we mentioned earlier, PRS combines the elastic network model with linear response theory to compute the residue fluctuation profile of an unbound conformation upon exerting a random external force on a residue, and it is shown to be very successful in capturing binding-induced conformational changes [39]. When a ligand approaches a receptor, it exerts forces around binding pockets, inducing certain dynamical changes. Here, we utilize PRS to mimic the nature of a binding event by exerting forces on the binding sites of an unbound conformation. Thus, we analyze the residue response fluctuation profile upon exerting random forces on binding sites of unbound conformations and identify the residues showing distinctive responses (i.e. higher fluctuation than the average fluctuation response) upon perturbing the residues at the binding sites. (See Materials and Methods for details.)

Elastic network models (ENMs) are utilized to explore allosteric behaviors in proteins [43]–[51]. ENMs are based on a purely mechanical approach, viewing a protein structure as an interconnected series of springs between interacting residue pairs. They provide information on equilibrium fluctuations and the various contributions to those fluctuations from different modes of motion. Moreover, by introducing a specific perturbation to the system and measuring its *dynamic* response, ENMs can provide detailed information about the energy landscapes, beyond the correlations between equilibrium fluctuations. To this aim, there are new modified ENMs developed whereby perturbations are introduced through modifying effective force constants [49], [50], distances between contacting pairs [52], or both [45]–[46]. Most of these analyses are focused on changes in the most functionally related mode (i.e. usually the slowest modes) upon perturbations. Although an ENM approach itself, our PRS model differs in two aspects. First, we introduce perturbations by inserting random external forces on the nodes of unbound conformations, (i.e. α-carbons) instead of modifying the distances between pairs of nodes or spring constants. This enables us to exert external forces on the binding sites (i.e., random Brownian kicks) and analyze the residues affected by the perturbation on the binding sites similar to the natural allosteric regulations where an approaching ligand induces certain dynamical changes in distal parts of the protein. Second, PRS uses the entire Hessian matrix to compute the residue displacement response upon exerting random forces on the selected residues. The allosteric regulation in small domain proteins like PDZs can arise through changes in dynamics [11], [14], unlike large conformational changes observed in large systems such as GroEL [47], [50] and myosin [53]. Therefore, more than one normal mode can contribute to allosteric regulations. In that respect, the advantage of using the full Hessian matrix in PRS can induce several related modes upon perturbation at the binding site.

### Identification of critical residues in allosteric regulation of PDZ interactions

**Figure 1:**
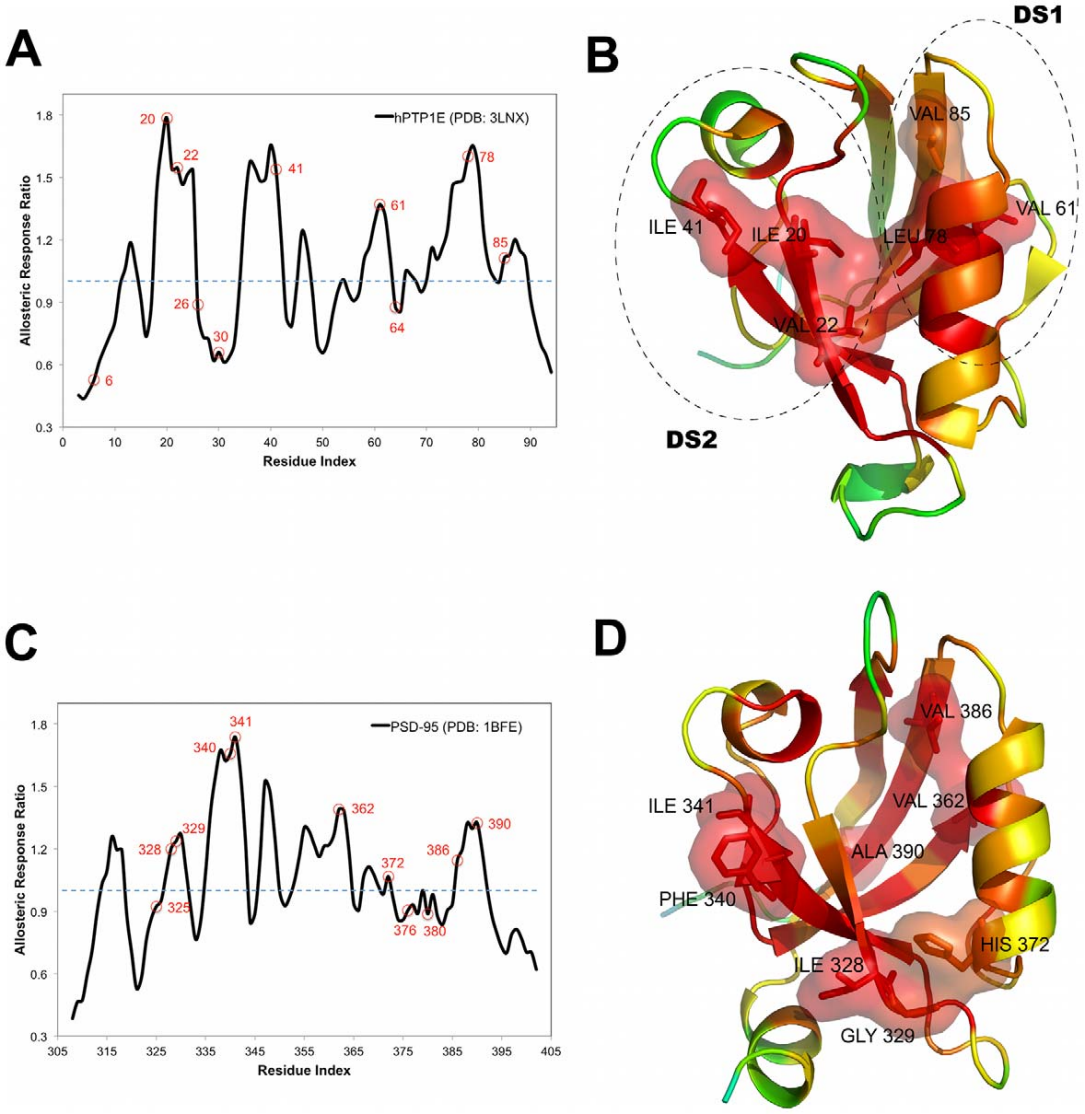
The allosteric response ratio profiles and ribbon diagrams of hPTP1E and PSD-95. The allosteric response ratio plots as a function of residue index for (A) hPTP1E PDZ2 (PDB entry: 3LNX) and (C) PSD-95 PDZ3 (PDB entry: 1BFE) along with the ribbon diagrams colored with respect to allosteric response ratio profiles (B and D). The key residues obtained from recent experimental studies are illustrated with red dots in these plots. The residues that give the highest mean square fluctuation response upon perturbation of binding pocket residues from PRS are displayed in the corresponding ribbon diagrams. The residues whose perturbation leads to a high response (χ_i_>1.00 for hPTP1E and PSD-95) are red, whereas residues with a low response are shown in blue within a color spectrum of red-orange-yellow-green-cyan and blue. The residues that match with experimentally determined ones are shown in stick representation. In hPTP1E PDZ2, distal surface 1 (DS1) contains residues in the N terminal of β6 and the anti-parallel β strand formed by β4 and β5 (Val61, Val64 and Val85) and distal surface 2 (DS2) located next to helix α1 consists of residues Val40 and Ile41. The figures were drawn using PYMOL [67].

Mutagenesis and NMR relaxation methods demonstrated that a network of residues exists that has a dynamic response upon ligand binding in both hPTP1E PDZ2 and PSD-95 PDZ3 [12], [19], [20], [25], [36]. Thus, we applied our approach to the unbound structures of two PDZ domain proteins: hPTP1E (PDB entry: 3LNX) and PSD-95 (PDB entry: 1BFE) and computed the allosteric response ratio χ*_j_* for each residue, which is the normalized average mean square fluctuation response of residue *j* upon perturbing only the binding site residues over the mean square average response of the same residue *j* obtained by perturbations on all residues. Thus, the index of allosteric response ratio χ enables us to identify residues that are more sensitive to perturbation around the binding pocket. **Figure 1** presents the allosteric response ratio profiles of (A) hPTP1E and (C) PSD-95 and the corresponding color-coded ribbon diagrams of these two proteins. Experimentally identified residues are marked with red dots. The ribbon diagrams of (B) hPTP1E and (D) PSD-95 are colored based on the allosteric response ratio, χ*_j_*, using a spectrum of red (the highest mean square fluctuation response) to orange, yellow, green, cyan and blue (the lowest response). The residues with the highest allosteric response ratio (χ*_j_*>1.00) are shown as stick representations. Particularly, those in agreement with the experimental analysis are labeled. Overall, there is a good agreement with experimentally identified allosteric residues and those predicted by our approach. Using χ*_j_*>1.00 as a threshold value for the allosteric response ratio, we predicted 6 out of 10 experimentally identified allosteric residues for hPTP1E [25] and similarly 8 out of 11 for PSD-95 [19] (i.e. the predicted residues correspond to the peaks in the allosteric response ratio profiles). We would like to note that we also tested our approach in another allosteric PDZ domain, SAP97 (PDB entry: 2AWX) which shows slight conformational change upon binding [18]. Using the same threshold value for χ*_j_*>1.00, we were able to distinguish not only the residues near canonical binding sites but also those distant from the binding site (Table S1), indicating the predictive power of PRS in identifying allosteric residues.

To our knowledge, all of previous computational studies including all-atom molecular dynamics [31], [32] and the rotamerically induced perturbation method (RIP) [11] identified certain critical residues using the previous NMR structure of hPTP1E (See Table S2 for predictions based on the previous NMR structure by different methods). Here, we apply our computational approach to the recently reported high-resolution crystal structure of hPTP1E PDZ2 [25], indicating that new bound and unbound structures deviate from previously determined NMR structures of hPTP1E and there are very minor structural changes in PDZ2 upon peptide binding.

The previous study of the RA-GEF2 peptide binding to hPTP1E PDZ2 using NMR relaxation technique identified residues that have significant changes in their side-chain dynamics upon peptide binding [20], [36]. This study also revealed that there are two distal surfaces physically linked to the peptide-binding site: (i) “distal surface 1 (DS1)”, which contains residues in the N terminal of β6 and the anti-parallel β strand formed by β4 and β5 (Val61, Val64, Leu66, Ala69, Thr81, and Val85), and (ii)”distal surface 2 (DS2)”, located next to helix α1, consisting of residues Ala39 and Val40. In the recent study Zhang et al. [25] identified 10 residues (Ile6, Ile20, Val22, Val26, Val30, Ile41, Val61, Val64, Val78, Val85) that have significant changes in side-chain dynamics upon binding both RA-GEF2 and APC peptides to PDZ2. These identified residues overlap with the findings of their previous study and they are located in the region of the binding site (Ile20, Val22, Val26 in the β2 strand, and Leu78 in helix α2), DS1 (Val61, Val64 and Val85), and in DS2 (Ile41). The highest allosteric response ratios obtained by PRS are also observed for the same residues except Val26 and Val64 (Figure 1A). Other residues that give high mean square fluctuation response (χ*_j_*>1.0) are summarized in more detail in **Table 1**, and those which agree with the experimentally identified ones [25] are highlighted in boldface. We also construct a two-way contingency table that presents the pattern matching between the experimentally identified residues and our prediction by PRS using a Fisher's exact test. The resulting p-value of hPTP1E, 2.9E-2, from the test indicates that there is a statistically significant matching between experiment and our method (Table S4).

**Table 1:** Residues that give the highest mean square fluctuation response (χ*_j_*>1.00 for hPTP1E and PSD-95) upon perturbation by PRS analysis.

Protein	Hot Residues
**hPTP1E**	
PRS* based on apo structure (PDB entry = 3LNX)	11, 13, Ser17, 18-19, **Ile20**, 21, **Val22**, 23-25, 34-40, **Ile41**, 45-46, 58-60, **Val61**, 66, 69, 71, 73-77, **Leu78**, 79-81, **Val85**, 87
**PSD-95**	
PRS* based on apo structure (PDB entry = 1BFE)	314, 316, 326-327, **Ile328**, **Gly329**, 330, 335-339, **Phe340**, **Ile341**, 345-347, 353-356, 358-359, 361, **Val362**, 367, 370, **His372**, 375, 379, **Val386**, 387-389, **Ala390**

Residues shown in boldface agree with experimentally identified ones.

In addition, the residues critical in allosteric pathways are characterized via statistical coupling analysis (SCA) of an evolutionary network using a large and diverse multiple sequence alignment of the PDZ domain family. Using the SCA method, Lockless and Ranganathan [16] predicted a set of residues within the family of PDZ domains that communicate signals through the protein core. When we compare our predictions with those obtained from SCA, nine residues (Ser17, Ile20, Gly24, Gly25, Gly34, Ala46, Val61, His71 and Val85) emerge as the residues with high allosteric response ratio (χ*_i_)* that are in agreement with the evolutionary network residues of hPTP1E [16], [30], [54]. The Fisher' exact test based on our method and SCA provides a p-value of 5.0E-4, indicating a high level of agreement. (Table S4).

The residues identified with high allosteric response ratios for PSD-95 PDZ3 are also in good agreement with double mutant cycle analysis [19]. The two-way contingency table based on experiment and method resulted in a high level of pattern matching, with a Fisher's exact test p-value of 1.5E-3 (Table S4). The mutational study of Chi et al. [19] indicates that the three positions Gly329, Val362, and Ala376 yield significant energetic coupling interactions with His372. In fact, among these coupling interactions the interaction between His372 and Val362 show long-range energetic coupling in the PSD-95 PDZ3 domain. As shown in **Figure 1B**, PRS analysis also captures the importance of the long-range energetic coupling interaction between His372 and Val362 of the PSD-95 PDZ3 domain. In this context, it is worth noting that studies based on a non-equilibrium perturbation-based molecular dynamics technique, called anisotropic thermal diffusion (ATD) [22], and the rotamerically induced perturbation method (RIP) [11], [41], also reported a complete signaling pathway of PDZs including PSD-95. ATD analysis proposed a signaling pathway between His372 and Ile335 that passed through Ile327 and Phe325 [22]. RIP analysis has also shown that some PDZs have more dynamic responses than the others and this was highly coupled with evolutionary SCA analysis [11]. The general pattern derived from both perturbation based MD analyses agreed with that obtained from PRS (See details for Table S4). The list of residues identified as allosteric residues with these different methods for these two PDZs is presented in Tables S2 and S3.

Furthermore, the energetic coupling residues (Gly329, Leu323, Ile327, His372, Ala376, Gln384) in PSD-95 were also successfully identified using an ENM-based structural perturbation (SPM) method [33], [47], [49], [55] based on exploring the propagation of the response of a local perturbation at a given residue to all other residues in a given structure. As we mentioned earlier, the basic premise behind SPM and PRS methods is similar except the harmonic springs connected to residues are changed by a small amount in SPM whereas the force is directly applied to residues in PRS. In addition to that, SPM focuses on changes in the single mode upon perturbation. It is usually the 1^st^ slowest mode in large proteins [52]. However, in the case of the small domain protein of PSD-95, rather than the 1^st^ mode, the 13^th^ and 20^th^ slowest modes significantly overlap with binding induced fluctuations [33]. On the other hand, PRS does not use the bound structure. PRS uses the Hessian of the whole unbound conformation and it automatically includes the modes that induce a response vector upon exerting forces on the binding site residues.

### Allosteric pathways may differ between different PDZs due to local changes

By linking the residues involved in allosteric regulations with respect to their response behavior, we can construct the allosteric pathways with PRS. PRS enables us to measure the relative directionality between the responses of a pair of neighboring residues to a perturbation. (i.e. the alignment of their response vectors). If the residues collectively move in line, their directionality should be parallel. After obtaining the directionality of different pairs of residues, we carry out a systematic analysis of the residues with the highest allosteric response ratio. For these residues, we search all possible interactions with a window size of 3 and identify residue pairs that collectively move in line together. A pathway is constructed by linking the sequential pairs showing similar directional response upon perturbation. Each constructed pathway is weighted based on alignment angles (i.e. directional similarity) between linking residues. Then we select the pathway with maximum total weight.

**Figure 2:**
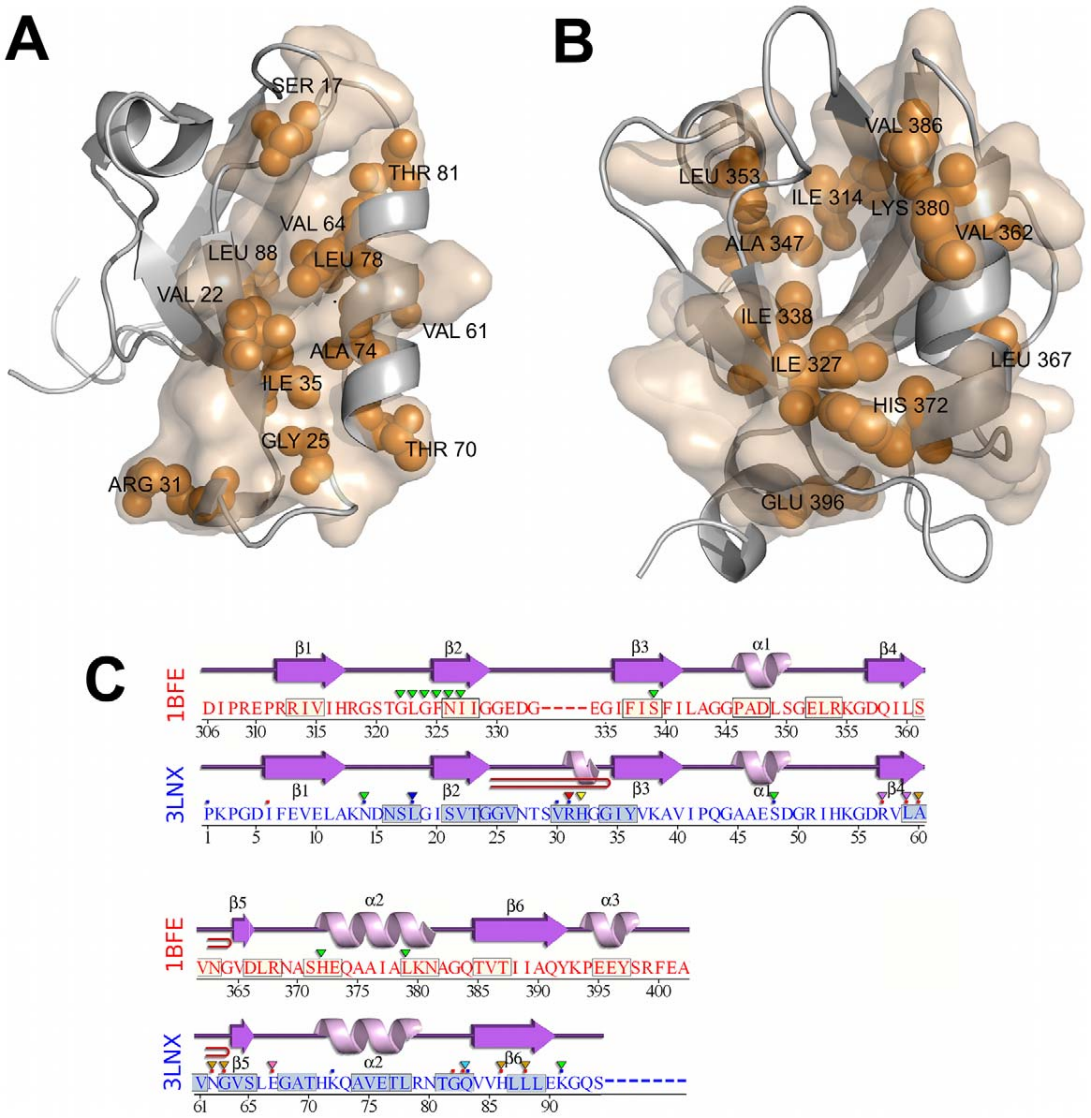
Intramolecular signaling pathways of hPTP1E and PSD-95 proposed by the PRS method. (A) The most highly weighted pathway of hPTP1E follows through connections Ser 17 → Val22 → Gly25 → Arg31 → Ile35 → Val61 → Leu64 → Thr70 → Ala74 → Leu78 → Thr81 → Leu88. The residues Val22, Ala39, Ile52, Val61 and Leu66 correspond to the residues in the dynamical network determined by experimental study. (B) The most highly weighted pathway of PSD-95 is obtained through connections Ile314 → Ile327 → Ile338 → Ala347 → Leu353 → Val362→ Leu367 → His372 → Ile380 → Val386 → Glu396. (C) Interestingly, these two pathways are clearly different; the predicted allosteric pathway of PSD-95 has a more homogeneous distribution through N-terminal to C-terminal, whereas the pathway of hPTP1E seems more localized especially in regions of β1-β2 loop, β2 and β3 strands and the region of β5 strand and the α2 helix. Identified residues in a window size of 3 for the pathway are highlighted in the sequence.

By this analysis, the allosteric pathway constructed for **hPTP1E PDZ2** follows through the connections Ser 17 → Val22 → Gly25 → Arg31 → Ile35 → Val61 → Leu64 → Thr70 → Ala74 → Leu78 → Thr81 → Leu88 (**Figure 2A**). Interestingly, the residues Val22, Val61, and Leu78 are located at the critical regions determined by the mutational analysis [25]. Since the model in the present study is low-resolution, we identify the residue Val22 that is near residue Ile20. The experimental mutational analysis showed that a change at Ile20 resulted in extensive changes in side chain dynamics while mutations at residues Ile35 and His 71 had a limited response in dynamics. Thus it is concluded that Ile20 might act as a hub that is energetically and dynamically important for transmitting changes in dynamics throughout the PDZ domain [36]. When we analyze the directionality preference of this residue with each residue identified for the most highly weighted pathway, we find that Ile20 collectively moves together with each of them, indeed acting as a hub in our dynamic network analysis. Moreover, the PRS pathway shows a remarkably high similarity (Ser17, Gly25, Ile35, Val61, His71, and Val75) with the statistical coupling analysis obtained by Lockless and Ranganathan [16].

As shown in **Figure 2B**, the most highly weighted pathway for **PSD-95** is obtained through connections Ile314 → Ile327 → Ile338 → Ala347 → Leu353 → Val362→ Leu367 → His372 → Lys380 → Val386 → Glu396. Interestingly, Val362 [16], [19], Lys380, and Val386 [16] yield significant energetic coupling interactions with His372 which are confirmed by mutagenesis studies. While the general pattern of signal propagation predicted from our method agrees with that inferred from the SCA analysis [16] there are some differences. The discrepancy between our model and the two proposed pathways by SCA may result because SCA analysis investigates the signaling pathway originating from a single residue, His372. However other residues at the binding pocket may be important for intramolecular signaling. Our analysis uses response profiles obtained by sequentially exerting a random force at a single residue along all the residues at the binding site. Thus, our approach might lead to the prediction of extra residues, such as Lys380, that interacts with the peptide and is near His372. Our model does not include Phe325 in the allosteric pathway, yet it finds Ile327, which is near residue 325. Moreover, MD analysis has shown that the mutation of Ile327 to Val leads to a dramatic signal reduction of Phe325, showing that position 327 is involved in mediating the signal pathway and highly linked with Phe325 [22].

Overall, when we compare the allosteric pathways of the two different PDZs, PSD-95 and hPTP1E, we see a clear difference (Figure 2C). There are some overlap regions between the two PDZ domains including residues in the β2 and β3 strands, the loop between β4 and β5 strands, and the C-terminal of the α2 helix. However, the predicted allosteric pathway of PSD-95 has a more homogeneous distribution through N-terminal to C-terminal, whereas the pathway of hPTP1E seems more localized, especially in regions of β1-β2 loop, β2 and β3 strands and the region of β5 strand and the α2 helix, missing the regions around the α1 helix. Indeed, the allosteric behavior of Ala347 in the α1 helix has also been found by SCA [16] and other MD analysis [22]. This comparison indicates that these two PDZs with similar sequences and structures have different allosteric behavior, indicating the role of dynamic coupling in single domain allostery. Thus, slight changes in the residue network changes dynamic coupling, which can lead to distinct allosteric paths.

### Local structural changes may lead to change in allosteric response

A recent experimental study [12] provided further support that allosteric communication can be driven by the network of residue interactions of PSD-95 without any conformational change. To investigate this phenomenon, they removed the non-canonical C-terminal third helix (α3, residues 394-399). Strikingly, removal lowers the binding affinity 21-fold and has a significant effect on the internal dynamics of PDZ3, even though it lies outside of the binding site and does not make direct interactions with the binding C-terminal peptide (CRIPT) residues.

**Figure 3:**
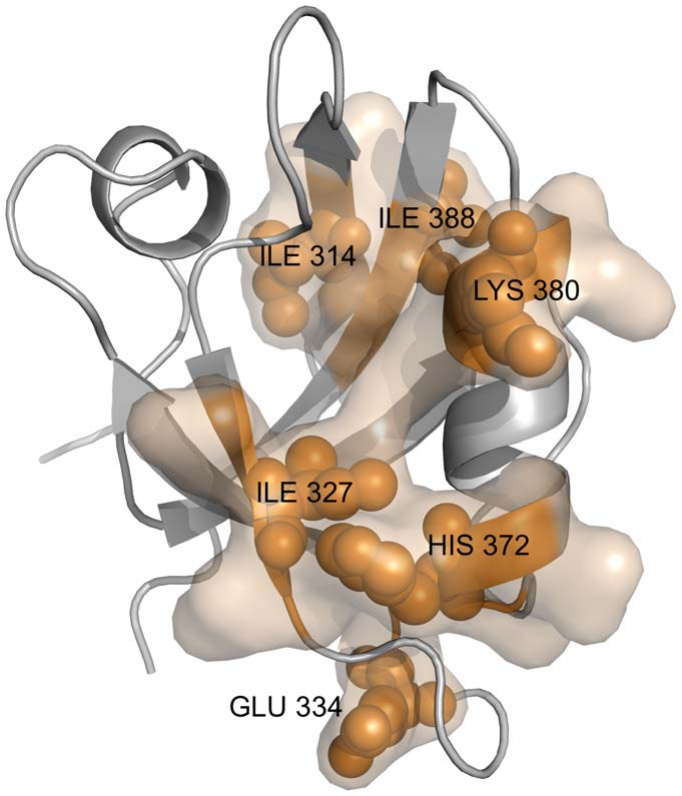
Intramolecular signaling pathway for PSD-95 with truncated third alpha helix predicted by the PRS method. We obtain the allosteric pathway for the truncated PSD-95 through connections Ile314 → Ile 327 → Glu334 → His372 → Ile380 → Ile388. However, the predicted allosteric pathway of full PSD-95 is different. The interactions specifically located in the α_1_ helix predicted for the PSD-95 were lost after removal of the α3 helix.

Using PRS, we also analyzed the truncated PSD-95 structure and investigated the impact of removal of helix α3 in the allosteric communication pathway. The most highly weighted pathway of the truncated structure is presented in **Figure 3**. Comparison of the pathway of PSD-95 (Figure 2B) and the truncated one (Figure 3) computed by PRS remarkably shows that the removal of the α3 helix significantly alters the allosteric pathway, indicating that the interactions responsible in transmitting intramolecular signals are being lost upon truncation of helix α3. For the truncated PSD-95 structure, the most highly weighted pathway has been identified through connections Ile314 → Ile 327 → Glu334 → His372 → Lys380 → Ile388, which is shown in **Figure 3**. Some of the interactions specifically located in the α1 helix and the loop between the β4 strand and the α2 helix predicted for the full PSD-95 were lost after removal of the α3 helix. Qian and Prehoda [37] showed that truncation of a portion of the α3 helix modulates and initiates the binding of Dlg to the localization protein GukHolder. Therefore, it is reasonable to say that this non-canonical α3 helix has a significant biological role in this allosteric regulation and the fact that the α3 helix is involved in the allosteric pathway obtained by PRS supports this.

In our recent work, we analyzed the dynamics of PDZs showing different binding specificities and showed that we can discriminate the binding specificity of PDZs based on their dynamics [35]. Within this picture, it is not surprising to see a change in binding affinity of PSD-95 upon truncation of the distal helix α3, because this leads to a change in dynamics. In order to investigate this any further, we also investigate the changes in the binding affinity upon removal of the α helix using docking techniques where we incorporate the changes in dynamics of PSD-95 into docking.

### Investigating the changes in the binding affinity upon removal of the helix α3 of PSD-95 using docking methodology

Computational docking methods are commonly used to identify the correct conformation of ligand-bound proteins along with their binding energy. However, docking algorithms predict incorrect binding modes or energies for about 50–70% of all ligands when the receptor is kept in a single conformation [56]. This is especially critical for PDZ whose dynamics play a key role in peptide binding specificity [35]. Some docking methods also incorporate the side chain flexibility of the receptor around binding pockets [57]–[60]. In our previous study [42], we incorporated the backbone flexibility of PDZs by generating multiple receptor conformations through restrained-replica exchange molecular dynamics (REMD) runs where the restraints are obtained by binding-induced elastic network modes. In this present study, we first generate multiple receptor conformations using the response vectors obtained upon perturbation of each residue via PRS. This provides us more computational efficiency in exploring conformational space. Then, we dock these multiple receptor conformations of PSD-95 and the truncated one against its native peptide (CRIPT) using RosettaLigand [58], [60]. RosettaLigand is docking software that computes the best-docked pose through a Monte Carlo minimization procedure in which the rigid body position and orientation of the small molecule and the protein side-chain conformations are optimized simultaneously. The lowest binding energy scores and corresponding peptide RMSDs of PSD-95 and the truncated third alpha helix of PSD-95 structures interacting with the CRIPT peptide are summarized in Table 2 for two different docking cases, (i) using only bound crystal structure (PDB code:1BE9) and (ii) using ensemble of structures obtained by applying PRS to the crystal structure. We cannot see this difference in binding affinities when we perform single receptor docking by using only the full and α3 helix truncated forms of the crystal structure. When we use PRS generated multiple receptor conformations to predict binding energies of PSD-95 and the truncated one, we find that the binding energy increases upon truncation of the C-terminal third alpha helix (α3 helix) as also observed experimentally [12]. This analysis indicates that the residue networks and their related dynamics indeed play a key role in binding affinities of PDZ. Our PRS analysis suggests that the significant change in the dynamics pathway of residue communication, caused by truncation of the α3 helix, leads to a change in binding affinity of its native peptide.

**Table 2:** Docking of native peptide (CRIPT) to bound structures and the best clustered one obtained from PRS.

	PSD-95			Truncated PSD-95		
Docking Approach	E_Rosetta_ (kcal/mol)	E_Drugscore_ (kcal/mol)	RMSD (Å)^a^	E_Rosetta_ (kcal/mol)	E_Drugscore_ (kcal/mol)	RMSD (Å)^a^
Single crystal structure	-13.69	-299.87	0.47	-12.06	-298.27	0.41
Ensemble docking with PRS	-16.35	-302.03	0.47	-14.84	-295.91	0.32

a RMSD values between the top scoring pose in Ångstroms measured over all heavy atoms of the peptide and the peptide's position in the crystal structure.

Allosteric responses in PDZs usually arise, because a perturbation at one site is transferred to the distal part of the protein through a network of residue communications. Here we investigate how the perturbation of a residue at the binding site is transferred through the dynamics of the residue network interactions. Thus we investigate the allosteric response of the two most investigated PDZs, PSD-95 and hPTP1E using our low resolution dynamics approach PRS. PRS is based on ENM where it uses only the topology of the given structure, and then using linear response theory, it computes the response fluctuation vector of each residue in the chain upon exerting a random force on a single residue. Using PRS, we compute the allosteric response ratio for each residue, which is the normalized average mean square fluctuation response upon perturbation. Most of the residues that are identified experimentally as residues in allosteric pathways indeed show high allosteric response ratios, indicating the consistency and usefulness of the PRS method for extracting the residues in the signaling pathway. Since PRS not only gives the mean square fluctuation of the response but also its directionality, we construct the allosteric pathway by linking the residues aligning in the same direction upon perturbations. Interestingly, our analysis has shown that the allosteric pathways of PSD-95 and hPTP1E are distinctively different from each other, despite the fact that they have similar structures. Likewise, we also observe a significant change in the allosteric pathway upon truncation of the distal α3 helix of PSD-95. Moreover, our flexible docking analysis where we generate an ensemble of multiple receptor conformations by PRS shows an increase in binding energy upon truncation. Overall, these results strongly suggest that local changes in residue network interactions can lead to changes in dynamics in allosteric regulations and various PDZs grasp to mediate different functions in the cell.

## Materials and Methods

### Benchmark

We analyze unbound structures of hPTP1E (3LNX) [25] and PSD-95 (1BFE) [61] in this study. The backbone root mean square deviation (RMSD) between hPTP1E and PSD-95 structures is 1.89 Å, while the sequence identity between pairs is only 36%. The all-atom RMSD between unbound and bound structures of PSD-95 is 1.13 Å (backbone RMSD = 0.73 Å) while that of hPTP1E is 1.03 Å (backbone RMSD = 0.46 Å).

### Perturbation Scanning Response (PRS) model

PRS is based on sequentially exerting directed random forces on single-residues along the chain of the structure and recording the resulting relative displacements of all the residues using LRT. The model views a protein structure as a three-dimensional elastic network. The nodes of the elastic network are Cα atoms of each residue where identical springs connect the interacting α-carbons in their native fold. In all elastic network models (ENMs), all residue pairs are subject to a uniform, single-parameter harmonic potential if they are located within an interaction range, or cutoff distance, *r_c_*. The major drawbacks of using cutoff distances are: (i) they are generally taken arbitrarily and (ii) their optimal values vary for different proteins [62], [63]. Instead of using any arbitrary cutoff distance, the interaction strength between all residue pairs can be weighted by the inverse of the square distance of their separation [63], [64]. We modify PRS by applying the concept of inverse square dependence for the interactions between residue pairs [63], [64] and introducing specificity between bonded and non-bonded interactions [35]. We tested the modified version on previously analyzed [39] 25 unbound protein structures that make various conformational changes upon bindings, and the results showed that the modified version successfully captures these conformational changes.

**Figure 4:**
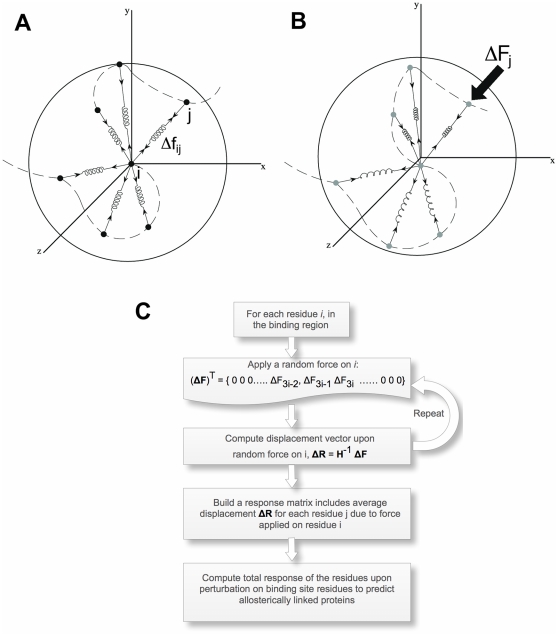
The Perturbation Scanning Response (PRS) method. (A) The free-body diagram of the central Cα atom of each sphere exhibits all of the pairwise interaction forces generated by the coordinating Cα atoms. In the absence of external forces acting on the system, each Cα atom must be in equilibrium under the action of interaction forces. (B) Under an external force applied on residue j, Δ**F**_j_, the residues change their original locations (shown in black dots in Figure 4A) in space. (C) Algorithm displaying the procedure used for predicting allosterically linked residues using PRS.

The free-body diagram of the central Cα atom of each sphere exhibits all of the pairwise interaction forces generated by the coordinating Cα atoms as schematically illustrated in **Figure 4A**. Each Cα atom must be in equilibrium under the action of interaction forces in the absence of external forces. The sum of forces on residue *i* along the x-, y-, and z-directions must be equal to zero under native state conditions,
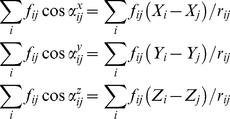
(1)where f_ij_ is the internal force on site i due to its interaction site *j*, 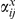,. is the angle between the x axis and the line of action of f_ij_, r_ij_ is the instantaneous separation vector between sites *i* and j and X*i*, Y*i* and Z*i* are the components of the instantaneous position, R*i*. The force balance can be generalized to the complete set of N sites (i.e. sites are Cα atoms of a protein) and M interactions (i.e. an interaction between any two Cα atoms is determined if the distance between two Cα atoms is less than the cut-off distance) as
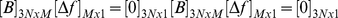
(2)where **B** is the directional cosine matrix.

If there are external forces acting on a set of residues of the folded structure as shown in **Figure 4B**, the force balance of the complete set of N sites and M interactions takes the following form:(DOC)
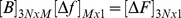
(3)where Δ**f** is the residual interaction forces and **ΔF** is a 3*Nx*1 vector containing the external force components at each residue. The native structure may undergo conformational changes about the equilibrium state under the action of these forces. During this process, the positional displacements **ΔR** and the bond deformations **Δr** are geometrically compatible. The relation between the positional displacement vector and the bond distance is given by
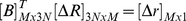
(4)where [**B**]^T^ is the transpose of **B**.

Within the scope of an elastic network of residues that are connected to their neighbors with springs, the interaction forces, Δ**f**, are related to the bond distance through Hooke's law by
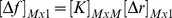
(5)where the coefficient matrix **K** is diagonal. Although the entries of **K** are taken to be equivalent in the original method [38], we introduce two different spring constants for the residue interaction network for bonded and non-bonded interactions, γ*_b_* and γ*_nb_*. The spring constant of the bonded part (γ*_b_*) is taken as 1. For the non-bonded part (γ*_nb_*), the interactions between residue pairs *i* and *j* are weighted by the inverse square of the distances, r_ij_ (as 8/r_ij_^2^).

Moreover, the work done by the external forces **ΔF** is equal to the work done by the internal forces **Δf** so substituting Equations (4) and (5) into Eq. (3), we obtain
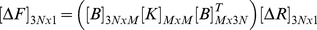
(6)


Let's note that the term 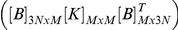 in Eq.(6) is also equivalent to the Hessian (**H**) [65].

On the other hand, one may choose to perturb a single residue or a set of residues, and calculate the response of the residue network through, 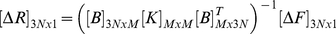
(7)or
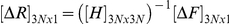
where the Δ**F** vector contains the components of the externally applied force vectors on the selected residues.

### Finding allosteric binding sites

In this study, first we apply a force as a unit vector on residue *i* along 7 directions (i.e. in x-, y-, z-, both x- and y-, both x- and z-, both y- and z-, all x, y, z directions. Then, we build a perturbation response matrix that includes average displacement ΔR for each residue *j* due to a force applied on residue *i*, 
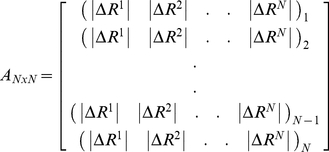
(8)where the magnitude of positional displacements for residue *j* in response to a perturbation at residue *i* is defined as,
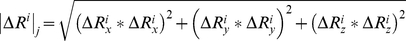
(9)


In order to predict which residues are critical in allosteric pathways, we distinguish the residues exhibiting significant fluctuation upon perturbation on binding site residues. Therefore, we define an index called the allosteric response ratio, χ*_j_* for each residue, which is the ratio of average fluctuation response of the residue *j* upon perturbations placed on binding site residues to average response of residue *j* upon perturbations on all residues, shown as:
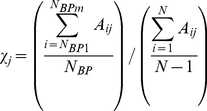
(10)where A_ij_ is the response fluctuation profile of residue *j* upon perturbation of residue *i*. The numerator is the average mean square fluctuation response obtained over the perturbation of the binding pocket (BP) residues, whereas denominator is the average mean square fluctuation response over all residue perturbation. Thus, N*_BP_* is the number of residues in the binding pocket and N*_BP1_* and N_*BPm*_ correspond to residue indexes in the binding pocket (residues 320-328 and 371-380 for PSD-95 and residues 16-23 and 70-79 for hPTP1E). To identify the critical residues in the allosteric pathway, for each residues we compute χ*_j_* in each perturbed direction and take into account of the maximum value of χ*_j_*. Then, we sort out all χ*_j_* and select the residue positions by setting a threshold of 1.0 or better. To understand how the sensitivity and specificity change, we predict the allosteric residues by varying the threshold of response ratio lower or higher than 1.00. We found that taking a threshold value lower than 1.0 gives same experimentally identified allosteric residues to ones obtained by using χ*_j_*>1.00 as a threshold value (Table S5).

We should note that the procedure has been also repeated using several random directions, rather than the 7 directions and we observed that our predictions do not change significantly. The schematic representation showing how we identify allosteric binding sites can be found in **Figure 4C**.

### Essential dynamics analysis

While PRS is a residue-based low-resolution approach, the essential dynamics analysis [66] is carried out on all-atom molecular dynamics (MD) trajectories to support the validity of the methodology. The details of the analysis are explained in Text S1. The comparison of residues that give the highest mean square fluctuation response (χ*_j_*>1.00 for PSD-95) upon perturbation with respect to the coarse-grained approach and the essential dynamics analysis is presented in Table S6. Overall, 82% of predicted residues from the essential dynamics analysis of all-atom MD trajectories are the same as those obtained by our low-resolution model (see Text S1 for more details). Moreover, the residues found by the coarse-grained approach that do not overlap with those of the all-atom approach are sequentially in close proximity to the residues identified by both approaches.

### Determination of pathways

PRS can be used to measure the degree of collectivity of the response of a group of neighboring residues to a perturbation on any residue. This enables us to construct an allosteric pathway through linking those residues showing similar response upon perturbations of the binding site.

To understand the nature of the response, the submatrix of residue *k* in response to perturbations in *i* from the inverse of the Hessian (See Equation 7) matrix can be decomposed into its eigenvalues and eigenvectors:
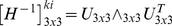
(11)


If the residues collectively move in line they have a single dominant eigenvalue and their corresponding eigenvectors should be parallel, indicating that they move cooperatively in the same direction. Therefore, to compare if the responses of two residues are same, we check the dot product of their corresponding eigenvectors,
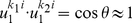
(12)where θ is the angle between the two eigenvectors. After obtaining the directionality of different pairs of residues upon perturbations on the binding site, we carry out a systematic network analysis using only the residues that give the highest fluctuation response upon perturbation. For these identified residues, we use a window size of 3 (i.e. if the residue 320 shows the highest mean square fluctuation response, the residues 319, 320, and 321 are taken into account), and search extensively to find residue pairs in sequence that move collectively upon perturbation. To this aim, we first calculate the overlap coefficients of the residue pairs by using the dot product of response vectors (Eq. 12). Using a cut off value of 0.98, we find the residue pairs that move in the same direction. Importantly, this means we identify the residue pairs showing also a high allosteric response ratio. We then perform an extensive search by generating all possible pathways through connecting these identified residue pairs and weight each pathway with the product of overlap coefficients. As an example, the predicted allosteric residue containing 314 in PSD-95 has the highest overlap coefficient with residue 327 with a value of 0.99. Then residue 327 has also very high overlap coefficient (with a value of 0.98) with residue 338. We then construct a pathway Ile314→Ile327→Ile338 which gives a total weight of 0.99x0.98 = 0.97. After exhaustive construction of all possible pathways we select the *pathway* with maximum total *weight*.

## Supporting Information

Table S1 The list of residues identified as the allosteric residues of SAP97 with the PRS and mutational analysis method.
(DOC)

Table S2 The list of residues identified as the allosteric residues of hPTP1E with different methods including statistical coupling analysis (SCA), molecular dynamics, rotamerically induced perturbations (RIP) and our PRS method.
(DOC)

Table S3 The list of residues identified as the allosteric residues of PSD-95 with different methods including statistical coupling analysis (SCA), the anisotropic thermal diffusion (ATD) method, the structural perturbation method (SPM), the rotamerically induced perturbations (RIP) method and our PRS method.
(DOC)

Table S4 Contingency tables showing a correlation between each method and experiments and each method and PRS for PSD-95 and hPTP1E.
(DOC)

Table S5 Residues that give the highest mean square fluctuation response when the threshold of response ratio is chosen lower/higher than 1.00.
(DOC)

Table S6 Residues that give the highest mean square fluctuation response (χ*_j_*>1.00 for PSD-95) upon perturbation by using coarse-grained approach and analysis based on all-atom REMD trajectories.
(DOC)

Text S1 Essential dynamics analysis.
(DOC)

## References

[1] Goodey NM, Benkovic SJ (2008). Allosteric regulation and catalysis emerge via a common route.. Nat Chem Biol.

[2] Monod J, Wyman J, Changeux JP (1965). On the Nature of Allosteric Transitions: a Plausible Model.. J Mol Biol.

[3] Koshland DE, Nemethy G, Filmer D (1966). Comparison of experimental binding data and theoretical models in proteins containing subunits.. Biochemistry.

[4] del Sol A, Tsai CJ, Ma B, Nussinov R (2009). The origin of allosteric functional modulation: multiple pre-existing pathways.. Structure.

[5] Gunasekaran K, Ma B, Nussinov R (2004). Is allostery an intrinsic property of all dynamic proteins?. Proteins.

[6] Hilser VJ (2010). Biochemistry. An ensemble view of allostery.. Science.

[7] Kern D, Zuiderweg ER (2003). The role of dynamics in allosteric regulation.. Curr Opin Struct Biol.

[8] Swain JF, Gierasch LM (2006). The changing landscape of protein allostery.. Curr Opin Struct Biol.

[9] Cooper A, Dryden DT (1984). Allostery without conformational change. A plausible model.. Eur Biophys J.

[10] Daily MD, Gray JJ (2007). Local motions in a benchmark of allosteric proteins.. Proteins.

[11] Ho BK, Agard DA (2010). Conserved tertiary couplings stabilize elements in the PDZ fold, leading to characteristic patterns of domain conformational flexibility.. Protein Sci.

[12] Petit CM, Zhang J, Sapienza PJ, Fuentes EJ, Lee AL (2009). Hidden dynamic allostery in a PDZ domain.. Proc Natl Acad Sci U S A.

[13] Popovych N, Sun S, Ebright RH, Kalodimos CG (2006). Dynamically driven protein allostery.. Nat Struct Mol Biol.

[14] Smock RG, Gierasch LM (2009). Sending signals dynamically.. Science.

[15] Tsai CJ, del Sol A, Nussinov R (2008). Allostery: absence of a change in shape does not imply that allostery is not at play.. J Mol Biol.

[16] Lockless SW, Ranganathan R (1999). Evolutionarily conserved pathways of energetic connectivity in protein families.. Science.

[17] Pan H, Lee JC, Hilser VJ (2000). Binding sites in Escherichia coli dihydrofolate reductase communicate by modulating the conformational ensemble.. Proc Natl Acad Sci U S A.

[18] Chi CN, Bach A, Engstrom A, Stromgaard K, Lundstrom P (2011). Biophysical characterization of the complex between human papillomavirus E6 protein and synapse-associated protein 97.. J Biol Chem.

[19] Chi CN, Elfstrom L, Shi Y, Snall T, Engstrom A (2008). Reassessing a sparse energetic network within a single protein domain.. Proc Natl Acad Sci U S A.

[20] Fuentes EJ, Der CJ, Lee AL (2004). Ligand-dependent dynamics and intramolecular signaling in a PDZ domain.. J Mol Biol.

[21] Gianni S, Walma T, Arcovito A, Calosci N, Bellelli A (2006). Demonstration of long-range interactions in a PDZ domain by NMR, kinetics, and protein engineering.. Structure.

[22] Ota N, Agard DA (2005). Intramolecular signaling pathways revealed by modeling anisotropic thermal diffusion.. J Mol Biol.

[23] Peterson FC, Penkert RR, Volkman BF, Prehoda KE (2004). Cdc42 regulates the Par-6 PDZ domain through an allosteric CRIB-PDZ transition.. Mol Cell.

[24] van den Berk LC, Landi E, Walma T, Vuister GW, Dente L (2007). An allosteric intramolecular PDZ-PDZ interaction modulates PTP-BL PDZ2 binding specificity.. Biochemistry.

[25] Zhang J, Sapienza PJ, Ke H, Chang A, Hengel SR (2010). Crystallographic and nuclear magnetic resonance evaluation of the impact of peptide binding to the second PDZ domain of protein tyrosine phosphatase 1E.. Biochemistry.

[26] Fan JS, Zhang M (2002). Signaling complex organization by PDZ domain proteins.. Neurosignals.

[27] Hung AY, Sheng M (2002). PDZ domains: structural modules for protein complex assembly.. J Biol Chem.

[28] Nourry C, Grant SG, Borg JP (2003). PDZ domain proteins: plug and play!. Sci STKE 2003.

[29] Lee J, Natarajan M, Nashine VC, Socolich M, Vo T (2008). Surface sites for engineering allosteric control in proteins.. Science.

[30] Suel GM, Lockless SW, Wall MA, Ranganathan R (2003). Evolutionarily conserved networks of residues mediate allosteric communication in proteins.. Nat Struct Biol.

[31] Dhulesia A, Gsponer J, Vendruscolo M (2008). Mapping of two networks of residues that exhibit structural and dynamical changes upon binding in a PDZ domain protein.. J Am Chem Soc.

[32] Kong Y, Karplus M (2009). Signaling pathways of PDZ2 domain: a molecular dynamics interaction correlation analysis.. Proteins.

[33] Liu Z, Chen J, Thirumalai D (2009). On the accuracy of inferring energetic coupling between distant sites in protein families from evolutionary imprints: illustrations using lattice model.. Proteins.

[34] De Los Rios P, Cecconi F, Pretre A, Dietler G, Michielin O (2005). Functional dynamics of PDZ binding domains: a normal-mode analysis.. Biophys J.

[35] Gerek ZN, Keskin O, Ozkan SB (2009). Identification of specificity and promiscuity of PDZ domain interactions through their dynamic behavior.. Proteins.

[36] Fuentes EJ, Gilmore SA, Mauldin RV, Lee AL (2006). Evaluation of energetic and dynamic coupling networks in a PDZ domain protein.. J Mol Biol.

[37] Qian Y, Prehoda KE (2006). Interdomain interactions in the tumor suppressor discs large regulate binding to the synaptic protein GukHolder.. J Biol Chem.

[38] Atilgan C, Atilgan AR (2009). Perturbation-response scanning reveals ligand entry-exit mechanisms of ferric binding protein.. PLoS Comput Biol.

[39] Atilgan C, Gerek ZN, Ozkan SB, Atilgan AR (2010). Manipulation of conformational change in proteins by single residue perturbations.. Biophys J.

[40] Vijayabaskar MS, Vishveshwara S (2010). Interaction energy based protein structure networks.. Biophys J.

[41] Ho BK, Agard DA (2009). Probing the flexibility of large conformational changes in protein structures through local perturbations.. PLoS Comput Biol.

[42] Gerek ZN, Ozkan SB (2010). A flexible docking scheme to explore the binding selectivity of PDZ domains.. Protein Sci.

[43] Bahar I, Lezon TR, Bakan A, Shrivastava IH (2010). Normal mode analysis of biomolecular structures: functional mechanisms of membrane proteins.. Chem Rev.

[44] Chennubhotla C, Yang Z, Bahar I (2008). Coupling between global dynamics and signal transduction pathways: a mechanism of allostery for chaperonin GroEL.. Mol Biosyst.

[45] Ming D, Wall ME (2005). Allostery in a coarse-grained model of protein dynamics.. Phys Rev Lett.

[46] Ming D, Wall ME (2005). Quantifying allosteric effects in proteins.. Proteins.

[47] Tehver R, Chen J, Thirumalai D (2009). Allostery wiring diagrams in the transitions that drive the GroEL reaction cycle.. J Mol Biol.

[48] Yang Z, Majek P, Bahar I (2009). Allosteric transitions of supramolecular systems explored by network models: application to chaperonin GroEL.. PLoS Comput Biol.

[49] Zheng W, Brooks BR, Thirumalai D (2006). Low-frequency normal modes that describe allosteric transitions in biological nanomachines are robust to sequence variations.. Proc Natl Acad Sci U S A.

[50] Zheng W, Brooks BR, Thirumalai D (2007). Allosteric transitions in the chaperonin GroEL are captured by a dominant normal mode that is most robust to sequence variations.. Biophys J.

[51] Zheng W, Brooks BR, Thirumalai D (2009). Allosteric transitions in biological nanomachines are described by robust normal modes of elastic networks.. Curr Protein Pept Sci.

[52] Zheng W, Brooks BR (2005). Probing the local dynamics of nucleotide-binding pocket coupled to the global dynamics: myosin versus kinesin.. Biophys J.

[53] Zheng W, Thirumalai D (2009). Coupling between normal modes drives protein conformational dynamics: illustrations using allosteric transitions in myosin II.. Biophys J.

[54] Socolich M, Lockless SW, Russ WP, Lee H, Gardner KH (2005). Evolutionary information for specifying a protein fold.. Nature.

[55] Zheng W, Brooks BR, Doniach S, Thirumalai D (2005). Network of dynamically important residues in the open/closed transition in polymerases is strongly conserved.. Structure.

[56] Totrov M, Abagyan R (2008). Flexible ligand docking to multiple receptor conformations: a practical alternative.. Curr Opin Struct Biol.

[57] Basdevant N, Weinstein H, Ceruso M (2006). Thermodynamic basis for promiscuity and selectivity in protein-protein interactions: PDZ domains, a case study.. J Am Chem Soc.

[58] Davis IW, Baker D (2009). RosettaLigand docking with Full Ligand and Receptor Flexibility.. J Mol Biol.

[59] May A, Zacharias M (2008). Protein-ligand docking accounting for receptor side chain and global flexibility in normal modes: evaluation on kinase inhibitor cross docking.. J Med Chem.

[60] Meiler J, Baker D (2006). ROSETTALIGAND: protein-small molecule docking with full side-chain flexibility.. Protein Sci.

[61] Doyle DA, Lee A, Lewis J, Kim E, Sheng M (1996). Crystal structures of a complexed and peptide-free membrane protein-binding domain: molecular basis of peptide recognition by PDZ.. Cell.

[62] Hinsen K, Petrescu AJ, Dellerue S, Bellissent-Funel MC, Kneller GR (2000). Harmonicity in slow protein dynamics.. Chem Phys.

[63] Yang L, Song G, Jernigan RL (2009). Protein elastic network models and the ranges of cooperativity.. Proc Natl Acad Sci U S A.

[64] Lin CP, Huang SW, Lai YL, Yen SC, Shih CH (2008). Deriving protein dynamical properties from weighted protein contact number.. Proteins.

[65] Atilgan AR, Durell SR, Jernigan RL, Demirel MC, Keskin O (2001). Anisotropy of fluctuation dynamics of proteins with an elastic network model.. Biophys J.

[66] Hayward S, de Groot BL, Kukol A (2008). Normal Modes and Essential Dynamics.. Molecular Modeling of Proteins.

[67] DeLano WL (2002). The PyMOL Molecular Graphics System..

