# Supplementary material for: Change in Allosteric Network Affects Binding Affinities of PDZ Domains: Analysis through Perturbation Response Scanning
Source: PLoS Comput Biol. 2011 Oct 6;7(10):e1002154. doi: 10.1371/journal.pcbi.1002154 (PMC3188487; doi:10.1371/journal.pcbi.1002154)
Supplement: Table S1 — The list of residues identified as the allosteric residues of SAP97 with the PRS and mutational analysis method. (DOC) [file pcbi.1002154.s001.doc]

**Table S1.** The list of residues identified as the allosteric residues of SAP97 with the PRS and mutational analysis method (Chi et al., Biochemistry 286, 3597-3606, 2011). We applied PRS to the unbound structure of SAP97 (2AWX), and identified the residues with high allosteric response ratio χ*_j_* > 1.00. The residues shown in boldface correspond to those identified experimentally. Highlighted residues represent a subset of residues that participate in noncanonical interactions either intradomain allostery or directly with residues on the ligand.

| **Protein** | **Hot Residues** |
| --- | --- |
| **SAP7** |  |
| PRS*  based on the unbound structure **(**PDB entry = 2AWX) | 319, 321, 323, **Lys324**, 327, **Gly328**, 329, **Gly330**, 331-334, **Gly335**, 345-350, **Thr351**, 352-353, **Gly356**, **Gly357**, 358-359, 364-366, 368-370, **Leu371**, 374, 376-378, 383, 386-392, **Asn393**, **Phe397**, 399 |
| EXPERIMENTAL  (Chi et al., Biochemistry 286, 3597-3606, 2011) | Group 1 (residues close to the canonical peptide-binding site): Lys324, Gly328, Gly330, Gly335, His341, Asn393  Group 2 (residues distant from the ligand binding site): Thr351, Ile354, Glu355, Gly356,  Gly357, His360, Leu371, Glu380, Glu385, Phe397 |
