# Supplementary material for: Change in Allosteric Network Affects Binding Affinities of PDZ Domains: Analysis through Perturbation Response Scanning
Source: PLoS Comput Biol. 2011 Oct 6;7(10):e1002154. doi: 10.1371/journal.pcbi.1002154 (PMC3188487; doi:10.1371/journal.pcbi.1002154)
Supplement: Table S2 — The list of residues identified as the allosteric residues of hPTP1E with different methods including statistical coupling analysis (SCA), molecular dynamics, rotamerically induced perturbations (RIP) and our PRS method. (DOC) [file pcbi.1002154.s002.doc]

**Table S2.** The list of residues identified as the allosteric residues of hPTP1E with different methods including statistical coupling analysis (SCA) (Lockless, Science 1999; Suel et al., Nature Struct. Biol. 2003), molecular dynamics (Dhulesia et al., J. Am. Chem. Soc. 2008; Kong and Karplus, Proteins 2008), rotamerically induced perturbations (RIP) (Ho and Agard, Protein Sci. 2010) and our PRS method. In the PRS analysis), the residues shown in boldface correspond to those identified experimentally and underlined residues represent those that are in agreement with the statistical coupling analysis (SCA).

| **Protein** | **Hot Residues*** |
| --- | --- |
| **hPTP1E** |  |
| PRS*  based on unbound structure (PDB: 3LNX) | 11, 13, 17, 18-19, **Ile20**, 21, **Val22**, 23, 24, 25, 34, 35-40, **Ile41**, 45, 46, 58-60, **Val61**, 66, 69, 71, 73-74, 75, 76-77, **Leu78**, 79-81, **Val85**, 87 |
| Experimental residues obtained upon binding of RA-GEF2 and APC  (Zhang et al., Biochemistry 2010) | Ile6, Ile 20, Val22, Val26, Val30, Ile41, Val61, Val64, Leu78, Val85 |
| Evolutionary Network by SCA (Lockless, Science 1999; Suel et al., Nature Struct. Biol. 2003) | Ser17, Ile20, Gly24, Gly25, Gly34, Ala46, Ile52, Val61, His71, Val75, Val85 |
| PRS*  based on unbound structure (PDB: 3PDZ) | 13, Ser17, Val22, Thr23, Gly25, 33, Gly34, Ile35, 36-38, Ala39, Val40, 41, 44, Ala45, Ala46, 47, Ile52, 53, 54-57, Val58, 59-60, Val61, 62, Leu66, His71, 74, Leu78, 79, Thr81, Val85, 86, Leu87, 88-89 |
| Experimental based on NMR structure- Dynamical Network (Fuentes et al., J. Mol. Bol. 2004; Fuentes et al., J. Mol. Biol. 2006) | Leu18, Ile20, Val22, Val26, Val30, Ala39, Val40, Val61, Val64, Leu66, Ala69, Leu78, Thr81, Val85 |
| Experimental - Dynamical Network –Extended (Fuentes et al., J. Mol. Bol. 2004; Fuentes et al., J. Mol. Biol. 2006; Dhulesia et al., J. Am. Chem. Soc. 2008) | Leu18, Ile20, Val22, Thr23, Val26, Val30, Ala39, Val40, Ala45, Ala46, Ile52, Val58, Val61, Val64, Leu66, Ala69, Leu78, Thr81, Val85, Leu87 |
| Structural Network – (Dhulesia et al., J. Am. Chem. Soc. 2008) | Leu18, Thr23, Asn27, Thr28, Ser29, Val30, Gly33, Ile35, Val37, Gln43, Ile52, His53, Lys54, Val58, Leu59, Asn62, Leu66, Leu78, His86, Leu87 |
| MD analysis (Kong and Karplus, Proteins 2009) | 3, Ser17, Leu18, 41, Gln43, 44, Ala45, Ala46, 47, 48, Val58, Leu59, Leu66, 67, 74, 90 |
| Rotationally Induced Perturbations (RIP)  (Ho and Agard, Protein Sci,  2010) | Leu11, Lys13, Leu18, Ile20, Ile35, Ala46, Ser48, Ile52, 56, Val58, Val61, Leu66, Ala74, Thr77, Leu78, Val85, Leu89 |
