# Supplementary material for: Change in Allosteric Network Affects Binding Affinities of PDZ Domains: Analysis through Perturbation Response Scanning
Source: PLoS Comput Biol. 2011 Oct 6;7(10):e1002154. doi: 10.1371/journal.pcbi.1002154 (PMC3188487; doi:10.1371/journal.pcbi.1002154)
Supplement: Table S3 — The list of residues identified as the allosteric residues of PSD-95 with different methods including statistical coupling analysis (SCA), the anisotropic thermal diffusion (ATD) method, the structural perturbation method (SPM), the rotamerically induced perturbations (RIP) method and our PRS method. (DOC) [file pcbi.1002154.s003.doc]

**Table S3.** The list of residues identified as the allosteric residues of PSD-95 with different methods including statistical coupling analysis (SCA) (Lockless, Science 1999; Suel et al., Nature Struct. Biol. 2003), the anisotropic thermal diffusion (ATD) method (Agard, J. Mol. Biol. 2005), the structural perturbation method (SPM) (Zheng et al., PNAS 2006), the rotamerically induced perturbations (RIP) method (Ho and Agard, Protein Sci. 2010) and our PRS method. In the PRS analysis, the residues shown in boldface correspond to those identified experimentally and underlined residues are those that are in agreement with the statistical coupling analysis (SCA).

| **Protein** | **Hot Residues*** |
| --- | --- |
| **PSD-95** |  |
| PRS*  based on unbound structure (PDB entry = 1BFE) | 314, 316, 326-327, **Ile328**, **Gly329**, 330, 335-339, **Phe340**, **Ile341**, 345-346, 347, 353, 354-356, 358-359, 361, **Val362**, 367, 370, **His372**, 375, 379, **Val386**, 387-389, **Ala390** |
| Experimental (Chi et al., PNAS 2008) | Phe325, Ile328, Gly329, Phe340, Ile341, Val362, His372, Ala376, Lys380, Val386, Ala390 |
| Statistical Coupling Analysis (SCA) | Gly322, Phe325, Gly329, Gly330, Ala347, Leu353, Val362, His372, Ala376, Lys380, Val386 |
| Anisotropic thermal diffusion (ATD) method | Phe325, Ile327, Ile341, Ala347, Leu353, His372 |
| Structural perturbation method (SPM) | Glu310, Arg318, Gly319, Ser320, Leu323, Ile327, Gly329, Glu331, Asp332, Gly333, Glu334, His372, Ala376, Lys380, Gln384, Phe400 |
| Rotamerically induced perturbations (RIP) method | 316, 318, 323, Phe325, 336, 346, Ala347, 349, Leu353, 357, 359, Val362, 367, 375, 378, 379, Val386, Ala390 |
