# Supplementary material for: Change in Allosteric Network Affects Binding Affinities of PDZ Domains: Analysis through Perturbation Response Scanning
Source: PLoS Comput Biol. 2011 Oct 6;7(10):e1002154. doi: 10.1371/journal.pcbi.1002154 (PMC3188487; doi:10.1371/journal.pcbi.1002154)
Supplement: Table S4 — Contingency tables showing a correlation between each method and experiments and each method and PRS for PSD-95 and hPTP1E. (DOC) [file pcbi.1002154.s004.doc]

**Table S4**. Contingency Tables Showing a Correlation^a^ between each method and experiments and each method and PRS for PSD-95 and hPTP1E.

| hPTP1E | PRS/Exp | significant | insignificant | Total |
| --- | --- | --- | --- | --- |
|  | significant | 8 | 31 | 39 |
|  | insignificant | 3 | 52 | 55 |
|  | total | 11 | 83 | 94 |
|  | ^a^ The *p*-value based on the Fisher’s exact test is 0.0285 | | | |

| hPTP1E | SCA/Exp | significant | insignificant | Total |
| --- | --- | --- | --- | --- |
|  | significant | 4 | 7 | 11 |
|  | insignificant | 7 | 76 | 83 |
|  | total | 11 | 83 | 94 |
|  | ^a^ The *p*-value based on the Fisher’s exact test is 0.0226 | | | |

| hPTP1E | PRS/SCA | significant | insignificant | Total |
| --- | --- | --- | --- | --- |
|  | significant | 10 | 29 | 39 |
|  | insignificant | 1 | 54 | 55 |
|  | total | 11 | 83 | 94 |
|  | ^a^ The *p*-value based on the Fisher’s exact test is 0.0005 | | | |

| PSD-95 | PRS/Exp | significant | insignificant | Total |
| --- | --- | --- | --- | --- |
|  | significant | 9 | 26 | 35 |
|  | insignificant | 2 | 60 | 62 |
|  | total | 11 | 86 | 97 |
|  | ^a^ The *p*-value based on the Fisher’s exact test is 0.0015 | | | |

| PSD-95 | SCA/Exp | significant | insignificant | Total |
| --- | --- | --- | --- | --- |
|  | significant | 7 | 4 | 11 |
|  | insignificant | 4 | 82 | 86 |
|  | total | 11 | 86 | 97 |
|  | ^a^ The *p*-value based on the Fisher’s exact test is 0.0001 | | | |

| PSD-95 | ATD/Exp | significant | insignificant | Total |
| --- | --- | --- | --- | --- |
|  | significant | 3 | 3 | 6 |
|  | insignificant | 8 | 83 | 91 |
|  | total | 11 | 86 | 97 |
|  | ^a^ The *p*-value based on the Fisher’s exact test is 0.0183 | | | |

| PSD-95 | SPM/Exp | significant | insignificant | Total |
| --- | --- | --- | --- | --- |
|  | significant | 4 | 12 | 16 |
|  | insignificant | 7 | 74 | 81 |
|  | total | 11 | 86 | 97 |
|  | ^a^ The *p*-value based on the Fisher’s exact test is 0.0801 | | | |

| PSD-95 | RIP/Exp | significant | insignificant | Total |
| --- | --- | --- | --- | --- |
|  | significant | 4 | 14 | 18 |
|  | insignificant | 7 | 72 | 79 |
|  | total | 11 | 86 | 97 |
|  | ^a^ The *p*-value based on the Fisher’s exact test is 0.1178 | | | |

| PSD-95 | PRS/SCA | significant | insignificant | Total |
| --- | --- | --- | --- | --- |
|  | significant | 10 | 25 | 35 |
|  | insignificant | 1 | 61 | 62 |
|  | total | 11 | 86 | 97 |
|  | ^a^ The *p* value based on the Fisher’s exact test is 0.0001 | | | |

| PSD-95 | PRS/ATD | significant | insignificant | Total |
| --- | --- | --- | --- | --- |
|  | significant | 5 | 30 | 35 |
|  | insignificant | 1 | 61 | 62 |
|  | total | 6 | 91 | 97 |
|  | ^a^ The *p* value based on the Fisher’s exact test is 0.0220 | | | |

| PSD-95 | PRS/SPM | significant | insignificant | Total |
| --- | --- | --- | --- | --- |
|  | significant | 2 | 33 | 35 |
|  | insignificant | 14 | 48 | 62 |
|  | total | 16 | 81 | 97 |
|  | ^a^ The *p* value based on the Fisher’s exact test is 0.0446 | | | |

| PSD-95 | PRS/RIP | significant | insignificant | Total |
| --- | --- | --- | --- | --- |
|  | significant | 12 | 23 | 35 |
|  | insignificant | 6 | 56 | 62 |
|  | total | 18 | 79 | 97 |
|  | ^a^ The *p* value based on the Fisher’s exact test is 0.0054 (p1=0.0037) | | | |
