# Supplementary material for: Change in Allosteric Network Affects Binding Affinities of PDZ Domains: Analysis through Perturbation Response Scanning
Source: PLoS Comput Biol. 2011 Oct 6;7(10):e1002154. doi: 10.1371/journal.pcbi.1002154 (PMC3188487; doi:10.1371/journal.pcbi.1002154)
Supplement: Table S5 — Residues that give the highest mean square fluctuation response when the threshold of response ratio is chosen lower/higher than 1.00. (DOC) [file pcbi.1002154.s005.doc]

We predict the allosteric residues using χ*_j_* > 1.00 as a threshold value for the allosteric response ratio. To understand how the sensitivity and specificity change, we predict the allosteric residues by varying the threshold of response ratio lower or higher than 1.00 in Table S4. When we choose the threshold of response ratio higher than 1.22 (i.e. corresponds to the 75 percentile of all allosteric response ratio data), we only predict 6 out of 11 experimentally identified allosteric residues for PSD-95. When the threshold of the response ratio decreases to 0.65 (corresponds to the 47 percentile of the data), we identify 8 out of 11 experimentally identified residues that are similar to ones obtained by using χ*_j_* > 1.00 as a threshold value. Moreover, the additional residues predicted with this cutoff are sequentially in close proximity to the residues predicted using χ*_j_* > 1.22 (i.e. we don’t predict new regions for the allostery). However, the total number of predicted residues increases when we decrease the response ratio to a threshold value of χ*_j_* > 0.65 so we find that 1.00 as an optimum value.

**Table S5.** Residues that give the highest mean square fluctuation response when the threshold of response ratio is chosen lower/higher than 1.00.

| **Protein** | **Hot Residues*** | **Total # of predicted residues** |
| --- | --- | --- |
| **PSD-95** |  |  |
| χ*_j_* > 1.22 (the 75 percentile) | 316, **Ile328**, 330, 335-339, **Phe340**, **Ile341**, 346-347, 353-356, 359, 361, **Val362**, 367, 379,**Val386**, 388-389, **Ala390** | 25 |
| χ*_j_* > 1.00 (the 65 percentile) | 314, 316, 326-327, **Ile328**, **Gly329**, 330, 335-339, **Phe340**, **Ile341**, 345-347, 353-356, 358-359, 361, **Val362**, 367, 370, **His372**, 375, 379, **Val386**, 387-389, **Ala390** | 35 |
| χ*_j_* > 0.65 (the 47 percentile) | 313-318, 323, 326-32, **Ile328**, **Gly329**, 330, 334-339, **Phe340**, **Ile341**, 342, 345-348, 350, 353-361, **Val362**, 363, 367-368, 370, **His372**, 375, 379, 384-385, **Val386**, 387-389, **Ala390**, 391, 397 | 52 |
