# Supplementary material for: Change in Allosteric Network Affects Binding Affinities of PDZ Domains: Analysis through Perturbation Response Scanning
Source: PLoS Comput Biol. 2011 Oct 6;7(10):e1002154. doi: 10.1371/journal.pcbi.1002154 (PMC3188487; doi:10.1371/journal.pcbi.1002154)
Supplement: Table S6 — Residues that give the highest mean square fluctuation response (χj>1.00 for PSD-95) upon perturbation by using coarse-grained approach and analysis based on all-atom REMD trajectories. (DOC) [file pcbi.1002154.s006.doc]

**Table S6.** Residues that give the highest mean square fluctuation response (χ*_j_* > 1.00 for PSD-95) upon perturbation by using coarse-grained approach and analysis based on all-atom REMD trajectories. Residues shown in boldface agree with experimentally identified ones. Highlighted residues are those predicted using both coarse-grained (ENM) and all-atom REMD analysis.

| **Protein** | **Hot Residues** |
| --- | --- |
| **PSD-95** |  |
| Based on coarse-grained approach | 314, 316, 326-327, **Ile328**, **Gly329**, 330, 335-339, **Phe340**, **Ile341**, 345-347, 353-354, 355, 356, 358-359, 361, **Val362**, 367, 370, **His372**, 375, 379, **Val386**, 387-389, **Ala390** |
|  |  |
| Based on All atom REMD trajectory analyses | **Phe325**, 326-327, **Ile328**, **Gly329**, 330, 335-339, **Phe340**, **Ile341**, 355, 356, 358-359, 361, **Val362**, 363, 365, 367, 370, 375, 379, 382, 385, **Val386**, 387-389, **Ala390**, 391 |
