# Supplementary material for: Change in Allosteric Network Affects Binding Affinities of PDZ Domains: Analysis through Perturbation Response Scanning
Source: PLoS Comput Biol. 2011 Oct 6;7(10):e1002154. doi: 10.1371/journal.pcbi.1002154 (PMC3188487; doi:10.1371/journal.pcbi.1002154)
Supplement: Text S1 — Essential dynamics analysis. (DOC) [file pcbi.1002154.s007.doc]

**Text S1. Essential Dynamics Analysis**

While PRS is a residue-based low-resolution approach, the essential dynamics analysis is carried out on all-atom molecular dynamics (MD) trajectories to support the validity of the methodology. To this aim, we performed a 5 ns Replica Exchange Molecular Dynamics (REMD). The unbound structure of PSD-95 (1bfe) is seeded as the initial structure to the (REMD) simulation with the temperature range spanning from 270K to 450K. Replica spacing is set such that the swap likelihood between replicas approaches 0.45. The AMBER96 force field with a GB implicit solvent model and a SA penalty term of 5 cal/mol A° are used. The converged mass weighted covariance matrix is determined by analyzing 3.5-4ns, 4-4.5ns, and 4.5-5ns windows of the trajectories of the lowest replica. In the analysis, each snapshot of the trajectory is first aligned and centered to remove the translations and rotations and then a matrix X_n_ is generated for each sampling window

$X_{n}=r_{n}-\left\langle r_{n}\right\rangle$ (1)

where **r_n_** are 3N dimensional position vectors the the < > denote a time average for a specific sampling window. Then, the covariance matrix of that sampling window, **C_n_,** is calculated by

$C_{n,n}=\left\langle\left\langle X_{n}\right\rangle\left\langle X_{n}\right\rangle^{T}\right\rangle$ (2)

From the covariance matrix, the matrix of eigenvectors (**V_n_**) and the matrix of eigenvalues (**Λ_n_**) are obtained to construct the Hessian matrix.

$C=V\Lambda V^{T}$ (3)

The residues with the highest mean square fluctuation responses are computed by averaging over the three different time frames through application the linear response theory. Table S1 presents a comparison of residues that give the highest mean square fluctuation response obtained from coarse-grained and all-atom MD analysis. Using the allosteric response ratio χ*_j_* > 1.00 as a threshold value in both analyses, we predict 8 out of 11 experimentally identified allosteric residues for PSD-95. Interestingly, all-atom MD analysis predicts Phe325 while it cannot identify His372. With our low-resolution approach, we can identify His372 including residues 326 and 327 (i.e. being nearby residue of the experimentally determined Phe325). Overall, 82% of predicted residues from the essential dynamics analysis are same with those obtained by our low-resolution model.
